# The characteristic patterns of neuronal avalanches in mice under anesthesia and at rest: An investigation using constrained artificial neural networks

**DOI:** 10.1371/journal.pone.0197893

**Published:** 2018-05-24

**Authors:** Erik D. Fagerholm, Martin Dinov, Thomas Knöpfel, Robert Leech

**Affiliations:** 1 Centre for Neuroimaging Sciences, King’s College London, London, United Kingdom; 2 The Computational, Cognitive and Clinical Neuroimaging Laboratory, The Centre for Neuroscience, The Division of Brain Sciences, Imperial College London, Hammersmith Hospital Campus, London, United Kingdom; 3 Centre for Neurotechnology, Institute of Biomedical Engineering, Imperial College London, South Kensington Campus, London, United Kingdom; Consejo Nacional de Investigaciones Cientificas y Tecnicas, ARGENTINA

## Abstract

Local perturbations within complex dynamical systems can trigger cascade-like events that spread across significant portions of the system. Cascades of this type have been observed across a broad range of scales in the brain. Studies of these cascades, known as neuronal avalanches, usually report the statistics of large numbers of avalanches, without probing the characteristic patterns produced by the avalanches themselves. This is partly due to limitations in the extent or spatiotemporal resolution of commonly used neuroimaging techniques. In this study, we overcome these limitations by using optical voltage (genetically encoded voltage indicators) imaging. This allows us to record cortical activity *in vivo* across an entire cortical hemisphere, at both high spatial (~30um) and temporal (~20ms) resolution in mice that are either in an anesthetized or awake state. We then use artificial neural networks to identify the characteristic patterns created by neuronal avalanches in our data. The avalanches in the anesthetized cortex are most accurately classified by an artificial neural network architecture that simultaneously connects spatial and temporal information. This is in contrast with the awake cortex, in which avalanches are most accurately classified by an architecture that treats spatial and temporal information separately, due to the increased levels of spatiotemporal complexity. This is in keeping with reports of higher levels of spatiotemporal complexity in the awake brain coinciding with features of a dynamical system operating close to criticality.

## Introduction

Neurons in the cerebral cortex interact synaptically with one another at long and short range. These interactions result in system-wide complex dynamics over a broad range of spatial and temporal scales [1]. A particularly striking feature of these cortical circuit dynamics is the spontaneous emergence of cascade-like events, known as neuronal avalanches, that are able to propagate across large regions of the cortex [2]. Prolonged observation reveals a relationship between the statistics of avalanche size (the number of neurons or cortical regions activated in a given avalanche) and global brain state, e.g. anesthesia [3], sleep [4] and focused attention [5]. Neuronal avalanches appear to be ubiquitous in the brain, having been observed across scales varying by several orders of magnitude, from single-cell [6] to whole-brain [7] recordings in humans, as well as in other species [8–10]. Neuronal avalanches are associated with cortical dynamics operating at criticality, effectively a tipping point between ordered and chaotic regimes, hypothesized to be functionally advantageous [11]. However, it is currently not known whether specific avalanches have any biological relevance. In this study we investigate the avalanche phenomenon by asking: a) whether there are characteristic activation patterns produced by avalanches across different brain regions and b) if so, how do these patterns change with cognitive state? Avalanche patterns have been studied in several contexts [12–16], including whole-brain patterns [17], as well as a recent investigation into the relationship between avalanche patterns and cognitive states [18]. The latter found that avalanches patterns repeat more often than would be expected by chance, vary according to brain region, and correlate with behavior as well as asleep/awake states. Other studies showed that the anatomical patterns of avalanche propagation are similar to those of resting-state networks [7, 19]. In this study we build upon these results by studying avalanche patterns at high spatiotemporal resolution using customized machine learning techniques. Specifically, we use optical voltage imaging data of stimulus-free spontaneous activity from mouse layer 2/3 pyramidal cell populations. This technique allows us to probe the avalanche phenomenon, with the advantage of being able to image an entire hemisphere at both high spatial (~30um) and temporal (~20ms) resolution [20].

We begin by training artificial neural networks (ANNs), which have shown recent notable success in neuroscience [21], to distinguish between maps of voltage signals recorded either within avalanches or within periods of quiescence (the time between avalanches). Specifically, we use Restricted Boltzmann Machines (RBMs) [22], followed by feed-forward neural networks (FFNNs). RBMs are a type of generative ANN model that permit unsupervised learning of features from unlabeled input data. RBMs have two layers–input and hidden–with the hidden layer typically (and in our case) being of smaller dimension than the input layer. After training the RBMs, the hidden layer node activations represent a lower dimensional/compressed representation of the input layer activations. This compact RBM representation is then used to initialize FFNNs with supervised learning to discriminate between avalanches and quiescence. To date, ANNs, including RBMs, have shown success in extracting features from complex neural datasets [23, 24]. However, the way in which these ANNs learn a given problem remains unclear, due in part to the complexity of the architectures involved [25]. We address this issue by placing prior constraints on the cortical information able to contribute to the training of each artificial neuron (AN) within each hidden layer of our ANNs. This increases the transparency of the models and thereby facilitates neurobiological interpretation of the results.

We construct a series of these constrained ANNs—each designed to examine a specific aspect of the patterns produced by avalanches, by using architectures with different levels of spatial and temporal complexity. Following the training and validation stages, we cluster the hidden layer activations produced in the testing stage for each ANN and project the cluster centroids back through the architectures to the input layers. This allows us to: a) create maps showing the way in which characteristic avalanches are encoded within the hidden layers, and b) identify the points of origin of the avalanches, together with their trajectories across the cortex for both the anesthetized and awake states.

## Materials and methods

### Animal preparation

Animal experiments were performed in accordance with the National Institutes of Health guidelines for animal research and were approved by the Institutional Animal Care and Use Committees of the RIKEN Wako Research Center (Japan). Animals were euthanized in accordance with the National Institutes of Health Guidelines for Euthanasia of Rodents Using Carbon Dioxide, as published in the Animal Research Advisory Committee Guidelines. Mouse embryos at E14.5 to E15.5 were electroporated in-utero with the pCAG-VSFP Butterfly 1.2 plasmid as previously described [26, 27], except that the electroporation procedure was repeated three times, resulting in expression of the voltage indicator Butterfly 1.2 in layer 2/3 pyramidal cells in a large part of one cortical hemisphere. The skull was thinned to form a cranial window over the left hemisphere and a head post was implanted in adult (2–6 months old) in-utero electroporated mice under surgical pentobarbital anesthesia [26, 27]. Mice recovered from surgery for at least 3 days before being re-anesthetized with pentobarbital (80 mg/kg i.p.) and being head-fixed via an implanted head post in a custom-made stereotaxic frame with body temperature maintained at 37°C by means of a feedback-controlled heat pad (Fine Science Tools, Japan).

### Voltage imaging

Dual-channel voltage imaging of VSFP Butterfly 1.2 was performed with the following optical filters (Semrock): FF01-483/32-25 for mCitrine excitation, FF01-542/27-25 for mCitrine emission, BLP01-594R-25 for mKate2 emission, FF506-Di03-25x36 as an excitation beamsplitter, FF593-Di03-25x36 as a detection beamsplitter [26, 27]. Image sequences of 60 s duration and ~60 s pauses were then acquired at a 50Hz frame rate, 320x240 pixel resolution, using custom acquisition macros in ImagePro 6.2. The first 10s of each image sequence were discarded to remove possible contribution from the effects of shutter noise and excitation light at the start of each imaging sequence. Independent measures of brain state were obtained by simultaneously recording electroencephalogram (EEG) activity using an electrode placed on the contralateral skull. Chest wall movements were monitored using an optoelectronic sensor.

All analyses were performed on two datasets from one mouse, the first of which was collected within the first 20 minutes following delivery of pentobarbital (anesthetized state). The second dataset was collected between 180 and 200 minutes following drug delivery, at a time when the animal had recovered from anesthesia and was showing spontaneous whisking behavior, but no gross body movement (awake, resting state). These two datasets contained 10 segments of image sequences for each of the 2 brain states, with each image containing ~19.5 ms of averaged voltage imaging activity. Each image contained 15,676 pixels, with each pixel capturing a projected cortical area of 33um × 33um. All data are freely available through the Open Science Framework (https://osf.io/k5myf/).

### Data preprocessing

All data were preprocessed using the Matlab Image and Signal Processing Toolbox (Mathworks), as described previously [3]. Briefly, fluorescence images of the genetically encoded voltage indicator were gain equalized and the optical signal representing population membrane voltage was extracted by dividing the mKate2 by the Citrine signal [26, 27]. Finally, the voltage image sequences were temporally band-pass filtered at 0.1–20 Hz.

### Avalanche detection

The optical data represent changes in membrane voltage averaged across tissue volumes mapped onto image pixels and averaged over the time during which each image was acquired (~19.5 ms for the 50 Hz frame rate used). A positive signal value of a given pixel indicates that a large portion of layer 2/3 pyramidal neurons are depolarized from resting membrane potential. This can be understood as a synchronized transition to the up-state of the cortical circuitry projected to the pixel. Based on this conceptualization of the data, voltage image sequences were transformed to a sequence of binary maps. To this end, we subtracted the means from every time series of pixel values and divided them by their standard deviations (z-scored). For each pixel, the time points at which a threshold set at +3 s.d. was crossed from below were flagged as transitions to up-states, with all following above-threshold time points being set to zero until the next threshold crossing [7]. This high threshold ensured that the neural networks were trained only with the most pronounced events, thereby minimizing the possibility of noise contributing to the resultant weight distributions.

It should be noted that, as opposed to running the avalanche detection algorithm on point-process data in this way, it is possible to use data in which a pixel is labeled as being in the ‘on’ state for as long as it remains threshold. However, we use the point-process approach because this (a) avoids ‘double counting’ pixels, which may result in misleading statistics, (b) allows tracking of a wave front of activity, which is arguably more relevant to describing cross-cortical communication than retaining already activated areas, (c) conforms with the methodology used in similar avalanche literature [7], as well as our previous work with the same data [3, 28].

Avalanche detection was performed on the point process sequences described above. A total of 2104 avalanches were included in the analyses. We defined avalanches as clusters of transitions into up-states. Voltage images were identified as occurring within an avalanche if the following two conditions were both satisfied: a) clusters consisting of >10 pixels were tagged as transitions into the up-state within a given image, and b) the same was true for the two preceding and two following images. These conditions ensured that avalanches had minimum durations of 100 ms and minimum sizes of 50 pixels. Avalanches that did not meet these requirements were excluded in their entirety. The only condition for avalanche propagation was temporal contiguity of clusters as in the original definition [2]. Images classified as avalanche-free were similarly only retained if preceded and followed by two avalanche-free images–referred to in this study as periods of quiescence.

### Data preparation

The images flagged as belonging to either avalanches or periods of quiescence in the anesthetized and awake states were each divided into 80/10/10 training/validation/testing splits. The single image input architectures (1 and 4) were trained with single voltage imaging frames and the dual image input architectures (2,3,5,6,7) were trained with sets of two consecutive voltage imaging frames. An equal number of avalanche and quiescence images were selected randomly from the training (anesthetized: 2051/920 and awake: 1055/469 time points for single/dual image input data–see Artificial neural networks below), validation (anesthetized: 184/80 and awake: 151/68 time points for single/dual image input data) and testing (anesthetized: 205/88 and awake: 116/52 time points for single/dual image input data) sets and used across all subsequent analyses.

The z-scored training sequences were temporally concatenated and only pixels contained within the motor (2938 pixels), somatosensory (3177 pixels), visual (1134 pixels) and retrosplenial (1093 pixels) cortices were retained at the previously selected avalanche and quiescence time points. Two pixels were eroded from the perimeters of the same hand-drawn cortical regions used in our previous work [3] in order to ensure that the neural networks would only learn from intra-cortical neural activity. The cortical regions (identified via whisking responses) allow us to interpret results in terms of known anatomical delineations. It should be noted that the size of the drawn cortical regions could affect the overall contribution of a given region to the analyses. However, since the regions were kept constant across analyses, size differences cannot explain differences between model architectures and between different brain states.

### Artificial neural networks

All machine learning processes were performed using the Matlab Statistics and Machine Learning Toolbox (Mathworks) and DeepLearnToolbox [29].

**Fig 1:**
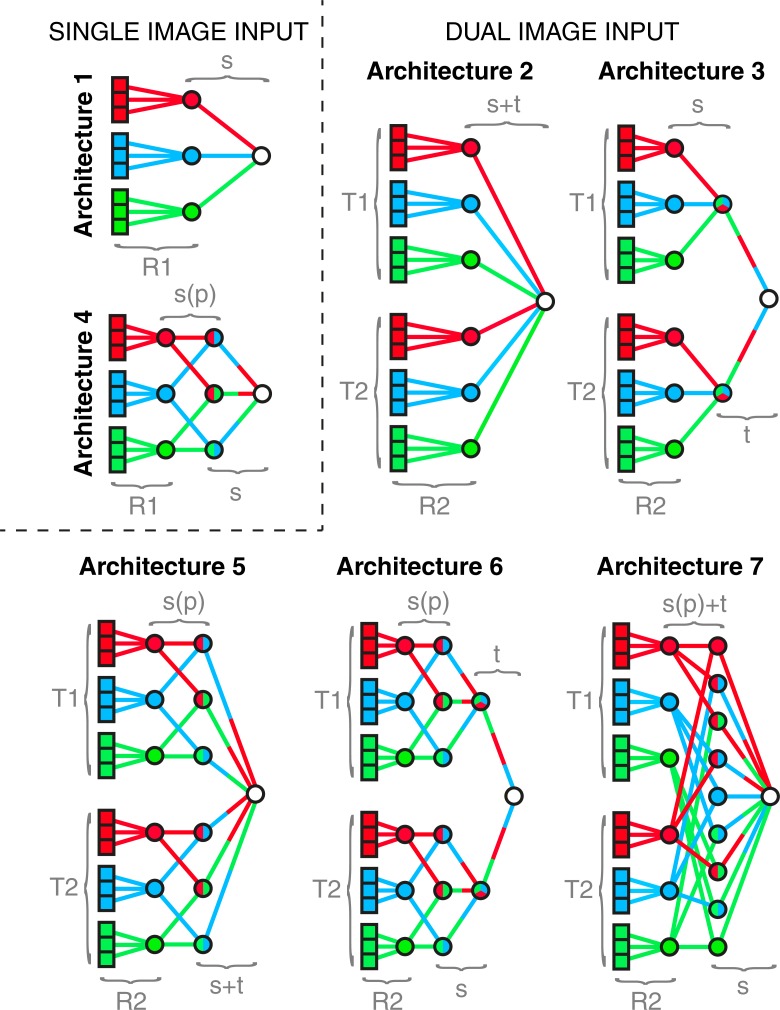
Illustrative models of the artificial neural network architectures used in this study. All illustrative architectures in this figure are based on a greatly simplified model of the cortex that consists of three regions (red, blue, green), each of which contains three pixels. Each AN in the first layer of every architecture corresponds to a cortical pixel—9 for single image input architectures (1 and 4) and 18 for dual image inputs architectures (2,3,5,6 and 7)—of which 9 are within the first (‘T1’) input image and 9 are within the second (‘T2’) input image. The weights between the input layer and first hidden layer of every architecture are initialized by previously trained RBMs (i.e. the pre-training)—‘R1’ for single image input architectures (1 and 4) and ‘R2’ for dual image input architectures (2,3,5 and 6). The first hidden layers of the single image input architectures (1 and 4) consist of 3 ANs, each of which is connected only to correspondingly colored ANs in their input layers. The first hidden layers of the dual image input architectures (2,3,5 and 6) consist of two sets of ANs, which connect only to their correspondingly colored ANs in the input layers, originating in the first (‘T1’) and second (‘T2’) input images, respectively. The final layer of every architecture consists of a single AN that is trained to activate when the input image lies within an avalanche. (Architecture 1) A schematic of the first and simplest architecture, in which the signals are not spatially mixed before layer 3. (Architecture 2) A schematic of the second ANN architecture, in which the signals are not spatially or temporally mixed before layer 3. (Architecture 3) A schematic of the third ANN architecture, in which the third layer consists of two ANs, the first and second of which are connected only to the ANs in the second layer that originate from ‘T1’ and ‘T2’, respectively. The signals are not spatially mixed before layer 3, and not temporally mixed before layer 4. (Architecture 4) A schematic of the fourth ANN architecture, in which the third layer consists of 3 ANs, each of which connects to a unique pair of ANs in the second layer. The signals become spatially mixed across pairs of regions (‘s(p)’) between layers two and then fully spatially mixed (‘s’) between layers three and four. (Architecture 5) A schematic of the fifth ANN architecture, in which the third layer consists of 6 ANs, the first and second half of which connect to a unique pair of ANs in the second layer that originates in ‘T1’ and ‘T2’, respectively. The signals become mixed spatially across pairs of cortical regions between the second and third layer (‘s(p)’) and then fully spatially and temporally mixed (‘s+t’) between layers three and four. (Architecture 6) A schematic of the sixth ANN architecture, in which the third layer consists of 6 ANs, the first and second half of which each connects to a unique pair of ANs in the second layer that originates in ‘T1’ and ‘T2’, respectively. The fourth layer consists of two ANs, the first and second of which are connected to the ANs in the third layer that originate in ‘T1’ and ‘T2’, respectively. The signals become mixed spatially across pairs of regions (‘s(p)’) between the second and third layer, fully spatially mixed (‘s’) between the third and fourth layer, and temporally mixed (‘t’) between the fourth and fifth layer. (Architecture 7) A schematic of the seventh ANN architecture, in which the third layer consists of 9 ANs, each of which connects to two ANs in the second layer, the first of which originates within ‘T1’ and the second within ‘T2’. The signals become mixed spatially across pairs of cortical regions as well as temporally (‘s(p)+t’) between the second and third layer and then fully spatially mixed (‘s’) between the third and fourth layer.

Seven different (gradient descent) ANN architectures are used in this study, broadly divided into two types that accept either single or dual images as input data. The single image architectures are shown by the illustrative models in Fig 1 (Architectures 1 and 4), and the dual image architectures by the illustrative models shown in Fig 1 (Architectures 2,3,5,6,7). The dual image inputs are labeled ‘T1’ and ‘T2’, where ‘T2’ is the time point immediately preceding ‘T1’. Note that the ‘T1’ frames are the same as those used for the single image architectures. We also explore the effect of constraining the spatial information sent between successive layers such as to only allow one unique pair of nodes in layer L to feed into each node in layer L+1. We label this type of spatial pairing constraint ‘s(p)’ in Fig 1, in contrast to the cases where there is a fully connected spatial mixture between subsequent layers, which we label ‘s’. The data contains four regions but for simplicity we illustrate the architectures with three regions in Fig 1. Note that the ‘s(p)’ constraint in the four-region data still refers to a mixing of region pairs. Note also that both T1 and T2 are entered as a single training image into neural networks that are agnostic with regard to the importance of the temporal ordering. Therefore, switching the order of T1 and T2 redistributes the weights so as to mirror this change, leaving the results invariant.

### RBM constraints

RBMs were constructed with the input layer consisting of one artificial neuron (AN) for every pixel in the four cortical regions and the hidden layer consisting of 40 ANs. The weights were constrained in such a way that the first 10 ANs in the hidden layer were connected only to pixels within the motor cortex with all other weights set to zero, resulting in those ANs receiving information only from the motor cortex. The same constraint was applied to the other three cortical regions and their corresponding sets of 10 ANs each in the hidden layer. Therefore, every weight connecting the two layers of the RBMs contained information only from a single cortical region, with all other weights being reset to zero upon completion of each training epoch.

### FFNN initialization

The anesthetized and awake training datasets were normalized between zero and unity and used to train separate RBMs (training epochs = 5103, α = 0.1) at values of momentum between 0 and 1 in steps of 0.1. The momenta that resulted in the lowest average reconstruction errors (S2 Table) were then used to retrain new RBMs with 5105 training epochs. These RBMs were used to initialize all single image input data FFNNs. The weights of the FFNNs were initialized to those of the corresponding previously trained RBMs. The batch sizes were set equal to the number of time points in the input data. This process was repeated with dual image input data, with optimum momentum values again first being established (S2 Table). These new FFNNs had output layers that contained 80 ANs. The first half of these were connected to the four cortical regions in the first image and the second half to the second image in the same constrained way as described above. Therefore, every weight in the dual image FFNNs contained information only from a single cortical region and at a single time point.

All FFNNs initialized by RBMs were feedforward backpropagation neural networks with logistic outputs and optimal inverse tangent activation functions of hidden layers. The learning rate was set to 2 (with a scaling factor 1), with sparsity target = 0.05, and L2 regularization = 0, non-sparsity penalty = 0 and dropout level = 0 across all ANNs [29].

All data used to train the FFNNs were identical to those used to previously train the RBMs. All FFNNs were trained with avalanche/quiescence binary classification (training epochs = 10^4^, α = 0.1) at values of momentum between 0 and 1 in steps of 0.1. The validation datasets were used to determine the values of momentum that resulted in the lowest mean-squared error values for each brain state and architecture (S1 Table). These optimum momentum values were then used with the testing datasets to produce all subsequent results. All spatial and temporal constraints on the architectures described below were applied in the same way as for the RBMs–i.e. by setting selected weights to zero upon completion of each training epoch.

### Mapping clustered hidden layer activations

#### k-means clustering

Activations in the hidden layers of the seven architectures described above were clustered (k-means++, k = 4) following feed-forward passes of the testing sets through the trained FFNNs. The value of k = 4 was chosen after running the same analyses with a) lower values of k, for which the clustering algorithm split the data into avalanche/quiescence time points that displayed subsets of the results obtained for k = 4, and b) higher values of k, for which the clustering algorithm split the data into avalanche/quiescence time points that displayed redundancies of the results obtained for k = 4. Every round of k-means clustering was repeated 10 times with a new set of initial centroids and the resulting model with the lowest sum of distances to all points used going forward. The clusters of hidden units for avalanche and quiescence periods in different brain states were then back-projected through the FFNNs and mapped onto the cortex to display intensity maps of the hidden layer activations.

#### Spatial entropy

The randomness of the intensity maps produced by the back-projected clustered hidden layer activity was characterized by their entropy, defined as −∑*plog*_2_*p*, where the probability, p, is obtained from histogram counts of all overlapping 10×10 neighborhoods around every pixel in the intensity maps.

#### Cortical activity maps

We also display the voltage imaging activity summed across the time points that were correctly clustered into avalanche and quiescence across all combinations of architectures and brain states. This allows us to gauge the relationship between the hidden layer maps and the input voltage imaging activity.

#### Avalanche trajectories

The trajectories of both avalanche types identified by the clustering algorithm in each brain state were estimated by plotting the direction of travel between the first and last connected cluster of the avalanches, i.e. directly from the thresholded (point-process) voltage imaging data.

## Results

### Overview of results

In the following section, we begin by determining which ANN architectures are able to classify avalanches detected in voltage imaging data with the lowest mean squared error (MSE), in both the anesthetized and awake states. The structures of these best-performing architectures allow us to infer how spatial and temporal elements of the data are combined for optimal classification in different brain states. We proceed by examining the weight distributions of these architectures to gain insight into the fine-grained patterns associated with the avalanche phenomenon. We then investigate the activity patterns encoded in the hidden layers by observing the ways in which they cluster into characteristic avalanche patterns across maps of the cortex. Finally, we relate these characteristic patterns to input cortical activity and extract the trajectories of the different types of avalanches identified by the clustering algorithm, for both the anesthetized and awake states.

### Classification accuracy and architectural complexity

**Fig 2:**
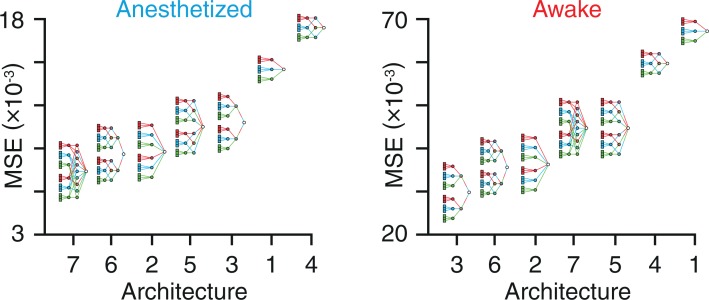
Classification accuracy and architectural complexity. Classification MSE for each of the seven architectures used in this study for the anesthetized and awake states. Architectures are ranked left to right with decreasing accuracy (increasing MSE) for both brain states.

The seven architectures used in this study are described in detail in Methods. These architectures are ranked here in order of the MSE associated with their ability to distinguish between frames of voltage imaging data, occurring either within avalanches or within periods of quiescence in the anesthetized and awake states (Fig 2).

#### Architecture ranking

Spatiotemporal elements of the data are mixed within the same layer in Architecture 7, achieving the lowest MSE in the anesthetized state. On the other hand, Architecture 3 achieves the lowest MSE in the awake state, using a model in which space and time are mixed one after the other in separate layers. In light of these results, we focus our analyses on three models going forward: a) Architecture 1, as it is the simplest model, which we treat as a reference, b) Architecture 3, as it allows for the lowest classification MSE in the awake state, and c) Architecture 7, as it allows for the lowest classification MSE in the anesthetized state. We then re-ran our analysis with avalanches defined as frames with at least 5 (instead of 10) active pixels and at least one other avalanche frame on either side (instead of two) and found that the optimum architecture did not change for either the awake or the anesthetized state. We found that the optimum architecture remained unchanged for both the anesthetized and awake states between avalanche thresholds of 2.6 and 3.7 s.d. Furthermore, we found that dilating the size of all cortical regions by five pixels left the optimum architecture unchanged.

#### Data lesioning

For completeness, we repeat all analyses with new datasets in which selected combinations of cortical regions are artificially lesioned. Lesioning in a combinatorially exhaustive manner allows us to determine to what degree a given region contributes to the power of a predictive model. Lesioning is performed only on the data used to train, validate and test the ANNs, but not on the data used for avalanche detection. Lesioning is performed in two ways for each cortical region. For example, in the case of the motor cortex, we: a) remove only the motor cortex from the data, and b) remove all regions except the motor cortex from the data. For each of these two lesioning methods, subsequent analyses are performed in two ways: a) by training, validating and testing with the lesioned data in anesthetized (S1 Fig) and awake (S2 Fig) states, and b) by validating and testing the lesioned data on ANNs that were previously trained and validated with non-lesioned data in anesthetized (S3 Fig) and awake (S4 Fig) states. The optimum momentum values obtained during the validation stages for all lesioned models are listed in S1 Table.

### Weight distributions

Here we report the characteristics of the weights connecting the layers of the architectures used, as this allows insight into which cortical regions and which time points were the most and the least influential in training the models. All weight distributions are reported as (mean ± s.d.) × 10^−2^. In the cases of architectures 3 and 7, values are reported as the mean of the temporally consecutive dual frame inputs.

#### Anesthetized state weight distributions

In the anesthetized state, the weights connecting the somatosensory cortex between the first and second layers have mean values close to zero in all three architectures and have low variability: Architecture 1 (0.2 ± 2.1), Architecture 3 (0.5 ± 1.7), and Architecture 7 (0.5 ± 1.7). The weights connecting the motor cortex between the first and second layers also have mean values close to zero, but in contrast to the somatosensory cortex, have high variability: Architecture 1 (2.2 ± 20.5), Architecture 3 (5.2 ± 18.3), and Architecture 7 (5.2 ± 18.2). In the case of the visual cortex, the weights between the first two layers are predominantly positive: Architecture 1 (69.9% positive), Architecture 3 (84.6% positive), and Architecture 7 (84.6% positive). Similarly, the weights between the first two layers connecting the retrosplenial cortex are predominantly positive in Architecture 1 (84.7% positive), Architecture 3 (80.4% positive), and Architecture 7 (80.0% positive).

#### Awake state weight distributions

**Fig 3:**
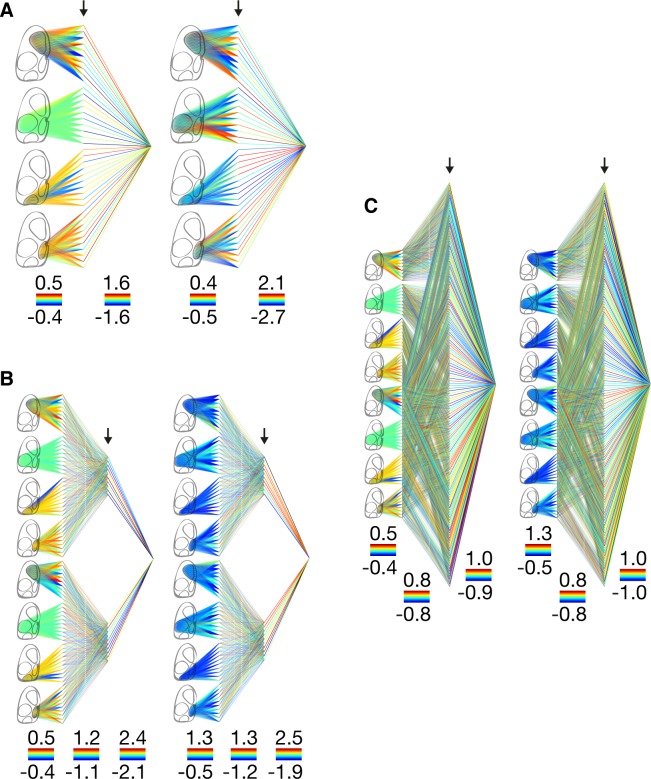
Weight distributions. (Architecture 1) Weight distributions in the anesthetized (left) and awake (right) states. Weights are normalized within each layer with hotter colors corresponding to higher weight values. The minimum and maximum weight values within each layer are indicated by the color bars shown below each layer. The arrows indicate the hidden layer in which the activations are subsequently clustered. (Architecture 3) Same layout as for Architecture 1. (Architecture 7) Same layout as for Architecture 1.

We observe a very different distribution in the awake state—the weights connecting the first and second layers in Architecture 1 are highly variable and have small negative mean values in the motor cortex (-4.0 ± 20.5), somatosensory cortex (-2.8 ± 19.2), and retrosplenial cortex (-3.2 ± 20.5). This is in contrast with the weights connecting the visual cortex, which are less variable and predominantly negative (-16.0 ± 11.7, 89.4% negative). The weights connecting the first and second layers are also predominantly negative across all cortices in Architecture 3 (66.8% negative) and Architecture 7 (66.7% negative) (Fig 3).

Weight distributions for the other architectures 2,4,5, and 6 are shown in parts (A) of S5, S6, S7 and S8 Figs, respectively.

#### Characteristic avalanche maps

**Fig 4:**
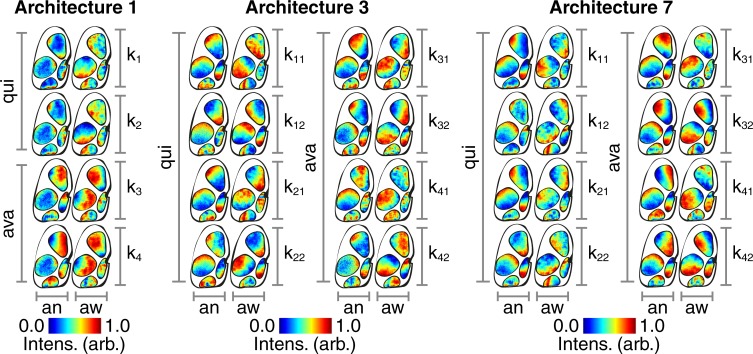
Hidden layer maps. (Architecture 1) Following clustering of hidden layer activations, the centroid locations are back-projected and mapped onto the mouse cortex. The clustering algorithm partitions the data into two types of quiescence (‘qui’, ‘k_1_’ and ‘k_2_’) and two types of avalanches (‘ava, ‘k_3_’ and ‘k_4_’) for the anesthetized (‘an’) and awake (‘aw’) states. Intensity values are normalized between zero and unity within each cortical region as indicated by the color bars. (Architecture 3) Same layout as for Architecture 1. (Architecture 7) Same layout as for Architecture 1.

Here we visualize the back-projected clustered hidden layer activations in the cortical space as a way of extracting biological significance from the way in which the various models encode information relevant to avalanche classification. There are two types of avalanches and two types of quiescence identified by the clustering algorithm. The way in which the ANNs encode these four states can be visualized by maps of back-projected clustered hidden layer activations (Fig 4).

**Fig 5:**
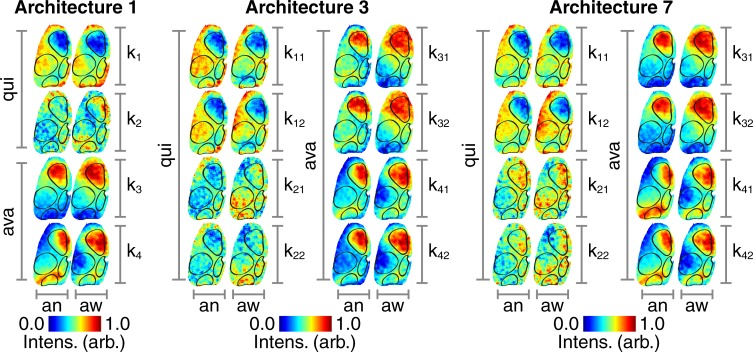
Cortical activity maps. (Architecture 1) Summed voltage images that are correctly separated into two types of quiescence (‘qui’, ‘k_1_’ and ‘k_2_’) and two types of avalanches (‘ava, ‘k_3_’ and ‘k_4_’) for the anesthetized (‘an’) and awake (‘aw’) states. Intensity values are normalized between zero and unity within each map as indicated by the color bars. (Architecture 3) Same layout as for Architecture 1. (Architecture 7) Same layout as for Architecture 1.

The cortical activity that results in these hidden layer activations is visualized by summing across the images that are correctly separated into their respective clusters (Fig 5). All intensity values are reported as (mean ± s.d.). In the cases of architectures 3 and 7, values are reported as the mean of the temporally consecutive dual frame inputs.

#### Cortical activity avalanche map characteristics

Both types of avalanches show a pronounced up-state across the motor cortex in Architecture 1 (anesthetized k_3_: 0.5 ± 0.3, anesthetized k_4_: 0.2 ± 0.3, awake k_3_: 0.6 ± 0.2, awake k_4_: 0.6 ± 0.2), Architecture 3 (anesthetized k_3_: 0.4 ± 0.3, anesthetized k_4_: 0.4 ± 0.3, awake k_3_: 0.6 ± 0.2, awake k_4_: 0.5 ± 0.2), and Architecture 7 (anesthetized k_3_: 0.5 ± 0.3, anesthetized k_4_: 0.2 ± 0.3, awake k_3_: 0.6 ± 0.2, awake k_4_: 0.4 ± 0.2). There are also signs of an up-state across the visual cortex that is most pronounced in the second avalanche type in the anesthetized state in Architecture 1 (k_4_: 0.3 ± 0.2) and Architecture 7 (k_4_: 0.3 ± 0.2).

#### Hidden layer avalanche map entropy in anesthetized vs awake states

The hidden layer maps have higher entropy in the awake state compared with the anesthetized state in both types of avalanches in Architecture 1 (k_1_: t(5190) = 97.02, p < 0.001, k_2_: t(5190) = 113.61, p < 0.001), Architecture 3 (k_1_: t(39,999) = 91.63, p < 0.001, k_2_: t(39,999) = 24.80, p < 0.001), and Architecture 7 (k_1_: t(119,999) = 84.28, p < 0.001, k_2_: t(119,999) = 14.76, p < 0.001).

#### Cortical activity quiescence map characteristics

The first type of quiescence shows a pronounced down-state across the motor cortex in Architecture 1 (anesthetized k_1_: -0.4 ± 0.3, awake k_1_: -0.6 ± 0.2), Architecture 3 (anesthetized k_1_: -0.4 ± 0.3, awake k_1_: -0.4 ± 0.2), and Architecture 7 (anesthetized k_1_: -0.4 ± 0.3, awake k_1_: -0.4 ± 0.2). There are also signs of a down-state across the visual cortex that is most obvious in the awake state of Architecture 3 (k_1_: -0.2 ± 0.2). The second type of quiescence, k_2_, shows a more diffuse distribution of up and down states without the cortical delineations seen in k_1_.

#### Hidden layer quiescence map entropy in anesthetized vs awake states

The hidden layer maps have higher entropy in the awake state compared with the anesthetized state in both types of quiescence, in Architecture 1 (k_1_: t(5190) = 58.49, p < 0.001, k_2_: t(5190) = 92.11, p < 0.001), Architecture 3 (k_1_: t(39,999) = 90.90, p < 0.001, k_2_: t(39,999) = 20.31, p < 0.001), and Architecture 7 (k_1_: t(119,999) = 47.90, p < 0.001, k_2_: t(119,999) = 65.96, p < 0.001).

#### Entropy in avalanche vs quiescence hidden layer maps

The hidden layer maps show higher levels of entropy across the quiescence states than the avalanche states in Architecture 1 (anesthetized: t(10,381) = 5.21, p < 0.001, awake: t(10,381) = 1.58, p < 0.001), Architecture 3 (anesthetized: t(79,999) = 57.50, p < 0.001, awake: t(79,999) = 67.81, p < 0.001), and Architecture 7 (anesthetized: t(239,999) = 53.26, p < 0.001, awake: t(239,999) = 75.08, p < 0.001).

Hidden layer and cortical activity maps for the other architectures 2,4,5, and 6 are shown in parts (B) of S5, S6, S7 and S8 Figs, respectively.

#### Avalanche trajectory maps

**Fig 6:**
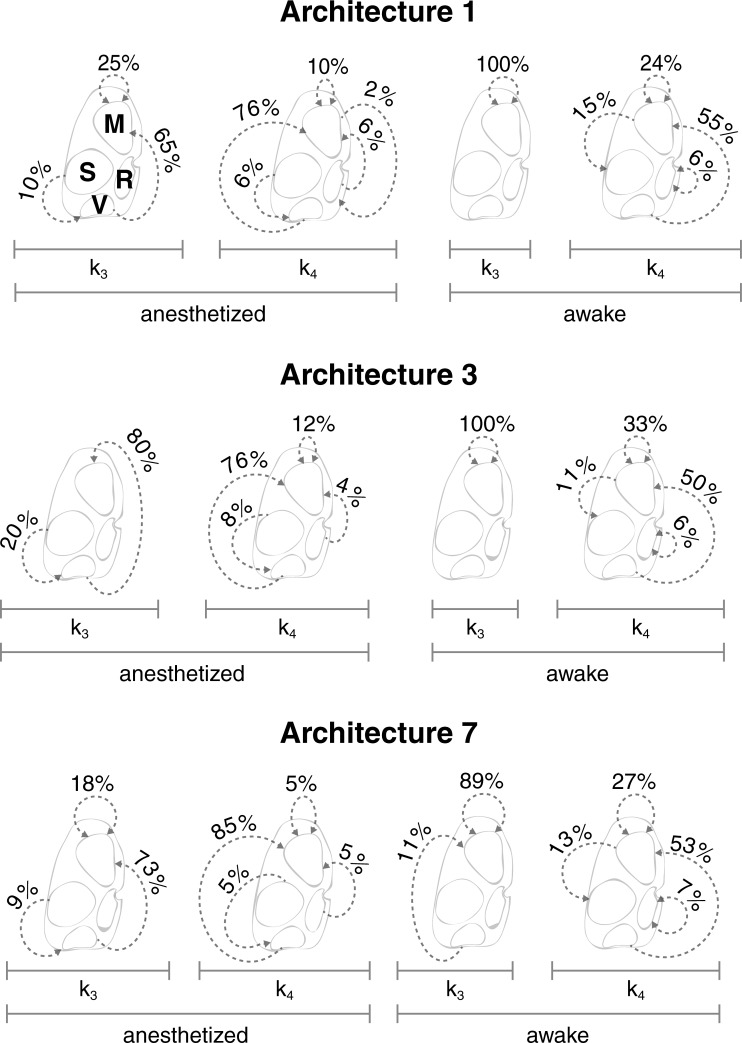
Avalanche trajectory maps. (Architecture 1) Dotted arrows represent the trajectories of avalanches, shown together with their rate of occurrence, for types k_3_ and k_4_ in the anesthetized and awake states using Architecture 1. (Architecture 3) Same layout as for Architecture 1. (Architecture 7) Same layout as for Architecture 1.

We next visualize the trajectories of the clustered avalanches in order to gain insight into the dynamics of their evolution and in order to assess the extent to which each type contributes to the pathways of cross-cortical communication. The trajectories of the two avalanche types (k_3_ and k_4_) are shown in Fig 6.

The dominant trajectories across all architectures originate in the visual cortex and terminate in the motor cortex, with the exception of k_3_ in the awake state, which consists of a unique (or dominant, in the case of Architecture 7) motor cortex loop, i.e. a trajectory that both originates and terminates within the motor cortex. The same kind of motor cortex loop appears less dominantly in all other examples, with the exception of k_3_ in the anesthetized state of Architecture 3. There is also an infrequent retrosplenial cortex loop that only appears in k_4_ in the awake state across all three architectures. It can also be seen that there are two trajectories that are unique to the anesthetized state across all three architectures. The first is a somatosensory-visual trajectory in k_3_ and the second is a retrosplenial-motor trajectory in k_4_. Similarly, there is a trajectory that is unique to the awake state that originates in the motor cortex and terminates in the somatosensory cortex in k_4_. The least frequent motor-retrospenial trajectory is picked up only in k_4_ by Architecture 1. It should be noted that the intensity of activity in the motor and visual cortices was not statistically different to the other cortical regions in either the anesthetized or awake states.

We show a movie of the first principle component of all visual-to-motor avalanches in the anesthetized state that last ten time points—the most common duration (S1 Video).

Avalanche trajectory maps for the other architectures 2,4,5, and 6 are shown in parts (C) of Supporting Information S5, S6, S7 and S8 Figs, respectively.

## Discussion

In this study, we explored the use of constrained ANNs to identify the characteristic patterns created by neuronal avalanches in the mouse cortex. The weights of the ANNs trained to identify these patterns provide insight into the spatial and, to some extent, the temporal structure of avalanches, as well as into the cortical regions involved. Below, we discuss the avalanche phenomenon from the perspectives of complex dynamical systems and neurobiology. We then evaluate the insights provided by our method of placing prior constraints on ANNs, as well as the potential of this approach to guide future research.

Local perturbations within complex dynamical systems can trigger cascade-like events that spread across significant portions of the system [30]. The size probability distribution of these cascades is indicative of the balance between local and global interactions. This cascade-like phenomenon has been observed to occur in the brain–referred to in relevant studies as neuronal avalanches. Studies of neuronal avalanches usually relate their findings to the statistics of large numbers of these events, without probing the characteristic patterns produced by the avalanches themselves. It is this point that we addressed by using machine learning techniques to encode the spatiotemporal patterns associated with different types of avalanches in mice studied in both anesthetized and awake states. We then projected the internal ANN representations onto maps of the mouse cortex to allow for a biological interpretation of the avalanche phenomenon.

To date it has not been possible to perform this type of analysis due to limitations in the experimental techniques available. Single-cell and local field potential recordings, for example, are not able to image a sufficient extent of the cortex. On the other hand, despite their ability to image at the whole-brain level, electrophysiological/functional techniques lack the required spatial/temporal resolutions, respectively. In this study however, we overcome these limitations by using optical voltage imaging using a genetically encoded voltage indicator that is selectively expressed in layer 2/3 pyramidal neurons to record cortical activity across an entire mouse cortical hemisphere *in vivo*, at both high spatial (~30um) and temporal (~20ms) resolution [26, 27].

Specifically, we used a series of ANNs to identify the characteristic patterns created by neuronal avalanches in the cortex of mice that were either in an anesthetized or resting awake state. The ANNs were trained to distinguish between images of average membrane voltage across local populations of layer 2/3 pyramidal cells at times when membrane depolarizations were either synchronized (avalanches) or desynchronized (quiescence). Seven ANN architectures were used, each designed to probe the avalanche phenomenon in a different way. This was achieved by constraining the weights to control which spatiotemporal elements of the data would subsequently be available during the training process. Following training and validation, the hidden layer activations of the ANNs obtained during the testing phase were clustered and back-projected through the ANNs to create intensity maps across the cortex. This allowed for visualization of how characteristic patterns associated with the avalanche phenomenon were encoded within each architecture.

In both the anesthetized and awake states the dual image input architectures (using temporally embedded data) outperformed the single image input architectures in distinguishing avalanches from quiescence. This is surprising, as the dual image input architectures contain a larger number of weights and therefore more degrees of freedom, rendering the problem harder to solve with the same amount of training data. Therefore, despite the increase in complexity of the relevant architectures, the avalanche phenomenon was best classified using two temporally consecutive images.

We found that the spatial entropy of the cortex was higher in the awake state compared with the anesthetized state, as predicted by theoretical accounts of self-organized criticality [31], and broadly in keeping with our recent work [28]. The avalanches in the anesthetized cortex were most accurately classified by an architecture in which spatial and temporal information was connected between successive layers. This was in contrast with the awake cortex, in which avalanches were most accurately identified by an architecture in which spatial and temporal information was connected separately between successive layers. These results indicate that it is advantageous to model the spatial and temporal features of awake cortical circuit dynamics separately, as these interact with one another in more complex ways than in the anesthetized brain. This result suggests a more complex pattern of both spatial and temporal information relating to cortical avalanches, which is broadly consistent with accounts of self-organized criticality in the awake brain. Future work with generative recurrent neural networks in which a ground truth is known may help us better characterize precisely why it is beneficial to model spatial and temporal information separately in the awake state. It should also be noted that these results may be subject to the same limitations reported in other studies, in which changes in noise correlations between brain states were shown to be a confounding factor [32].

It was found, across all seven architectures, that the weights connecting the somatosensory cortex from the input layer to the first hidden layer were all driven close to zero in the anesthetized state. This indicates that activity within the anesthetized somatosensory cortex was either irrelevant or detrimental with respect to distinguishing avalanches from periods of quiescence. However, the intensity of activity in the somatosensory cortex in the anesthetized state was not found to be statistically different to the other cortical regions in either the anesthetized or awake states. On the other hand, the mean of the weights across the motor cortex in the anesthetized state were also close to zero but their heterogeneity was higher than in the somatosensory cortex. This heterogeneity could reflect the variety of avalanche patterns that occur in the motor cortex. This is in contrast with the visual and retrosplenial cortices, in which the weights were predominantly positive in the anesthetized state, which could be indicative of a more predictable repertoire of neuronal avalanche patterns in these regions. However, there was no difference in either the variability of activity or in the spatial (image-wise) entropy between the motor and visual cortices. In the awake state, a more variable pattern of weights was observed across all four cortical regions. The reduction in the contribution of activity in somatosensory cortex to avalanches in the anesthetized state may reflect a reduction in sensory input. Throughout the analyses, the motor and visual cortices appeared to play important roles with respect to the identification of the avalanche state. Furthermore, the motor-motor and visual-motor avalanche trajectories were found to be the most frequent types as identified by the clustering algorithm.

Future research may be able to investigate avalanches defined with the additional constraint of spatial adjacency between temporally consecutive clusters [3]. This could also model dynamic states more fully by using recurrent neural networks [33] to better detect simultaneously occurring avalanches of different sizes and durations. Such lines of research may shed light on how to classify more complex behavior, such as the merging of multiple small avalanches into larger ones, or vice versa, the disintegration of large avalanches into smaller avalanches. A detailed exploration of these micro-events underlying what we observe as an avalanche could reveal clues as to the biological mechanisms underlying the phenomenon. Furthermore, it is possible that a fundamental description of the avalanche phenomenon could be obtained by extracting the form described within the hidden layers of ANNs. This would mean a significant step forward within theoretical neuroscience, as it would allow for the abstraction away from models, towards a mathematical expression of the behavior of neuronal avalanches.

## Supporting information

S1 Fig Full and lesioned models in the anesthetized state. A total of 43 architectures are shown, including the 7 described in Methods, together with the sub-architectures tailored to the following anesthetized training datasets: (a) 4 datasets, in each of which all but one cortical region have been lesioned, accounting for a total of 28 ANNs, and (b) 4 datasets, in each of which a single cortical region has been lesioned, accounting for just 8 ANNs in the table as only architectures 1 and 2 are meaningful for single region data. The letters on the left side of each cell correspond to the following: R—rank of architecture in question, in terms of mean squared error of testing data, A—architecture, with numbers corresponding to those in Fig 1. L—lesioned region(s), where the letters M,S,V,R correspond to the motor, somatosensory, visual and retrosplenial cortex, respectively, M—mean squared error (× 10^-4^) of the testing data. The figures in the middle of the cells are illustrative models showing which sections of the architectures are used in training, validation and testing, based on the architectures in Fig 1. The figures on the right of the cells show which cortical regions (in black) are used for each model.
(EPS)

S2 Fig Full and lesioned models in the awake state. Same layout as S1 Fig.
(EPS)

S3 Fig All models validated and tested with un-lesioned data in the anesthetized state. The 7 architectures described in Fig 1 are trained with the un-lesioned anesthetized datasets and then validated and tested using the same procedures described in S1 Fig.
(EPS)

S4 Fig All models validated and tested with un-lesioned data in the awake state. Same layout as S3 Fig.
(EPS)

S5 Fig Architecture 2. (Left) Weight distributions as described in Fig 3. (Right) Hidden layer maps (left) and cortical activity maps (right) as described in Figs 4 and 5, respectively. (Bottom) Avalanche trajectories as described in Fig 6.
(TIF)

S6 Fig Architecture 4. Same layout as S5 Fig.
(TIF)

S7 Fig Architecture 5. Same layout as S5 Fig.
(TIF)

S8 Fig Architecture 6. Same layout as S5 Fig.
(TIF)

S1 Table Optimum momentum values for all FFNNs. Optimum momentum values obtained in the validation stages. A = architecture, F = full model (non-lesioned), M = motor cortex, S = somatosensory cortex, V = visual cortex, R = retrosplenial cortex, Missing = models in which the given cortical region is lesioned, Single = models in which all cortical regions except the given cortical region are lesioned.
(XLSX)

S2 Table Optimum momentum values for all RBMs. A = architecture, divided into architectures that accept either single (1,4) or dual image inputs (2,3,5,6), with the remainder of the laid out in the same way as S1 Table.
(XLSX)

S1 Video Avalanche video. A video of the first principle component of all visual-to-motor avalanches in the anesthetized state that last ten time points (the most common duration), with superimposed vectors showing the direction of travel of the centre of mass between successive images. Once activated, a pixel remains colored in the video, for clearer visualization.
(MP4)
