# Supplementary figures and images for: The characteristic patterns of neuronal avalanches in mice under anesthesia and at rest: An investigation using constrained artificial neural networks
Source: PLoS One. 2018 May 24;13(5):e0197893. doi: 10.1371/journal.pone.0197893 (PMC5967741; doi:10.1371/journal.pone.0197893)

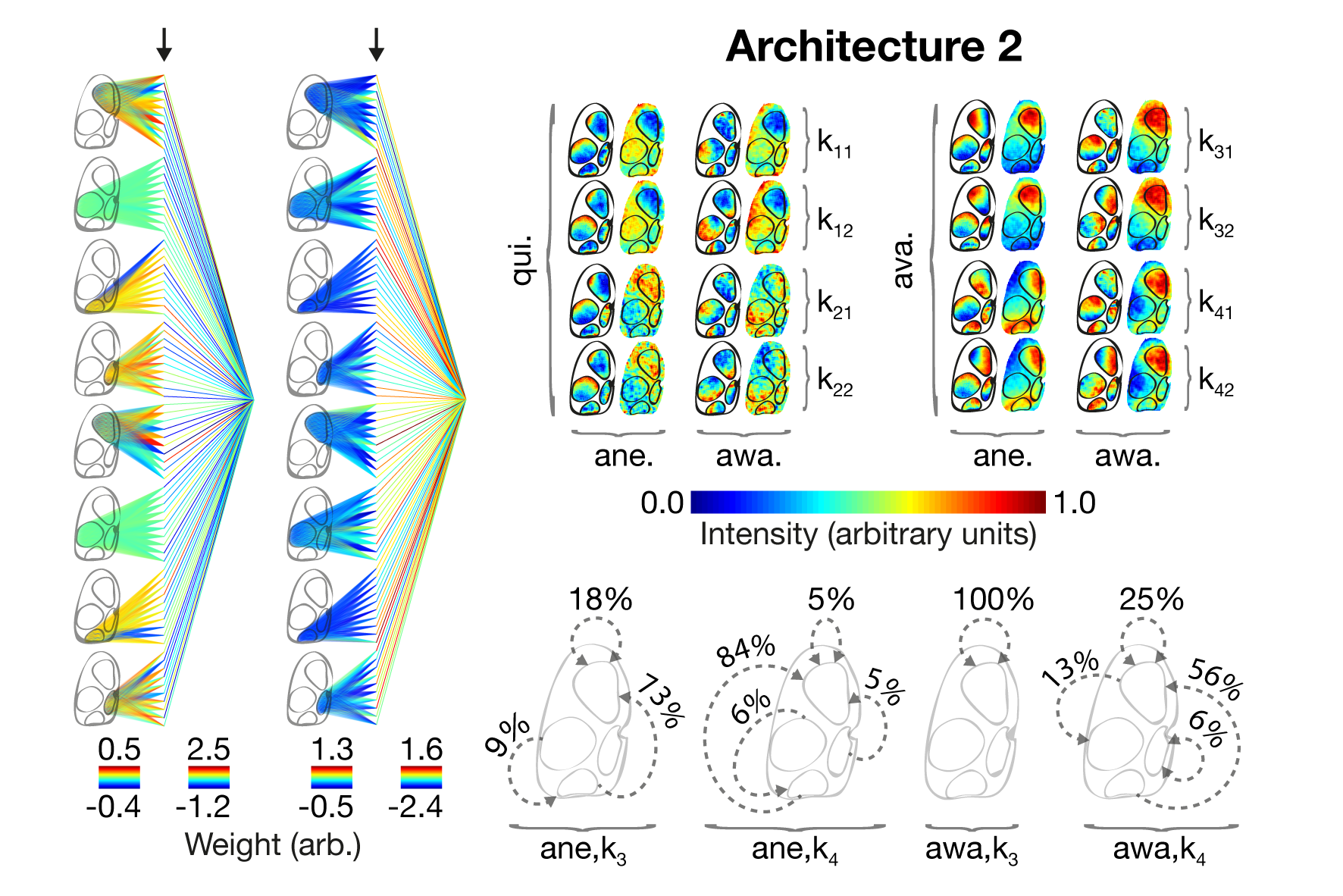

Supplement: S5 Fig — (Left) Weight distributions as described in Fig 3. (Right) Hidden layer maps (left) and cortical activity maps (right) as described in Figs 4 and 5, respectively. (Bottom) Avalanche trajectories as described in Fig 6. (TIF) [file pone.0197893.s005.tif]

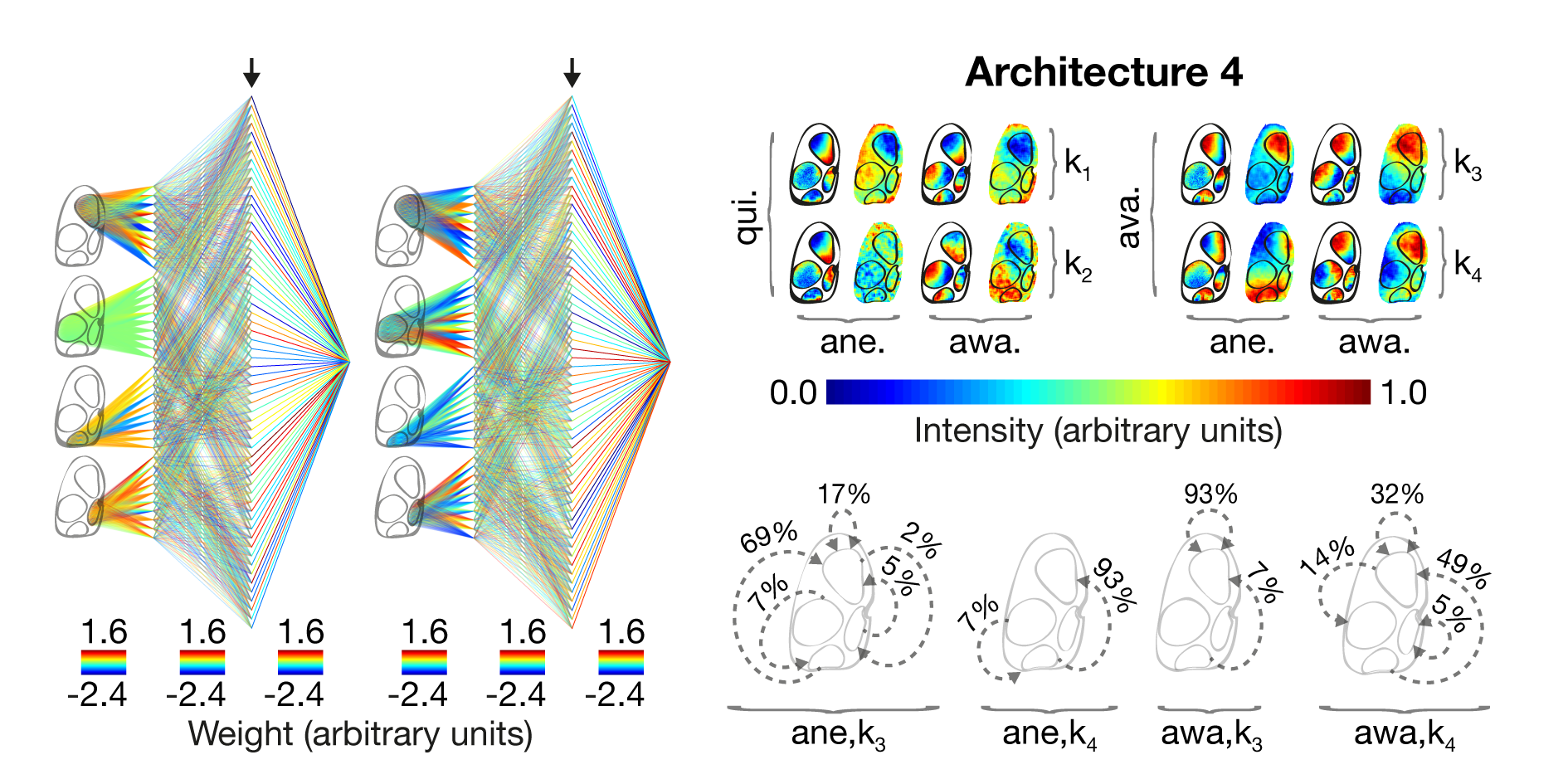

Supplement: S6 Fig — Same layout as S5 Fig. (TIF) [file pone.0197893.s006.tif]

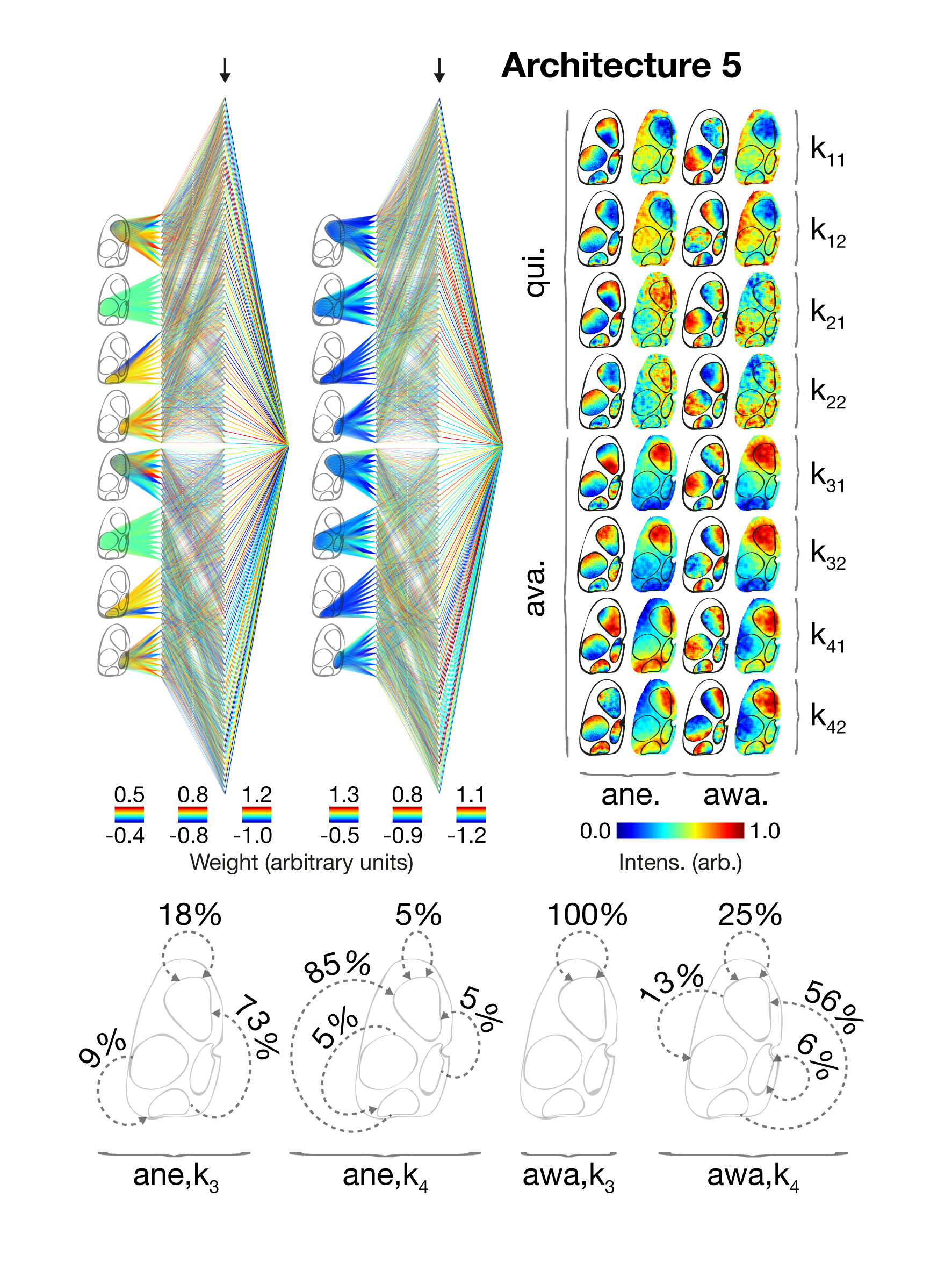

Supplement: S7 Fig — Same layout as S5 Fig. (TIF) [file pone.0197893.s007.tif]

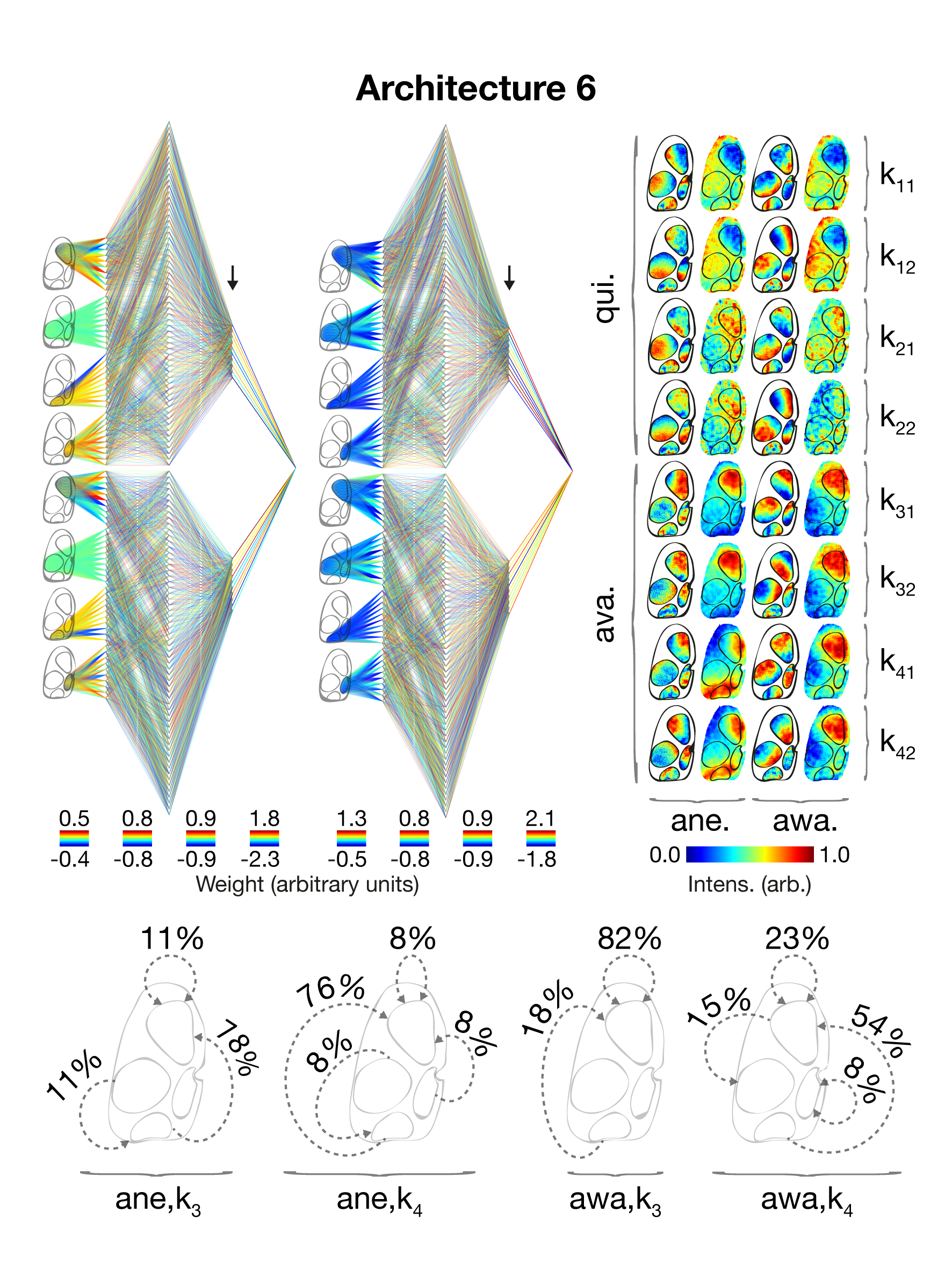

Supplement: S8 Fig — Same layout as S5 Fig. (TIF) [file pone.0197893.s008.tif]
